# Supplementary material for: Photo-reversible amyloid nanoNETs for regenerative antimicrobial therapies
Source: Nat Commun. 2025 Dec 10;16:11025. doi: 10.1038/s41467-025-65976-6 (PMC12696075; doi:10.1038/s41467-025-65976-6)
Supplement: Supplementary file 1 — Supplementary Information [file 41467_2025_65976_MOESM1_ESM.pdf]

Supplementary Information

Photo-reversible Amyloid NanoNETs for Regenerative Antimicrobial Therapies

Authors:

Qize Xuan[1,2]¶, Hui Li[1]¶, Yuan Gao[1]¶, Xinchi Qiao[1], Yifan Feng[1], Xinyu Yu[1], Jiazhe Cai[1], Tonghui Jin[2], Bin Liu[2], Mohammad Peydayesh[2], Jiaqi Su[2], Peter Fischer[2], Ping Wang[3], Chao Chen[1]*, Jiangtao Zhou[2,4,5,6]*, Raffaele Mezzenga[2,7]*

Affiliations:

[1]Institute for Environmental Pollution and Health, School of Environmental and Chemical Engineering, Shanghai University, Shanghai, 200444, PR China

[2]Department of Health Sciences and Technology, ETH Zürich, Schmelzbergstrasse 9, Zürich 8092, Switzerland

[3]Department of Bioproducts and Biosystems Engineering, University of Minnesota, St Paul, MN 55108, USA

[4]Department of Food Science and Technology, National University of Singapore, 2 Science Drive 2, 117542, Singapore

[5]Bezos Centre for Sustainable Protein at the National University of Singapore, 2 Science Drive 2, 117542, Singapore

[6]National University of Singapore (Suzhou) Research Institute, 377 Linquan Street, Suzhou Industrial Park, Jiangsu, 215123, China

[7]Department of Materials, ETH Zurich, Wolfgang-Pauli-Strasse 10, 8049 Zürich, Switzerland

¶These authors contribute equally.

*Corresponding authors:
chaochen@shu.edu.cn;
jtzhou@nus.edu.sg;
raffaele.mezzenga@hest.ethz.ch

Contents

Supplementary methods

Supplementary Fig. 1 | AFM images of flexible fibrils (FFs) at high resolution.

Supplementary Fig. 2 | Secondary structural characterization of RFs and FFs using CD spectra and ThT fluorescence intensity assays.

Supplementary Fig. 3 | Rheological characterization of RFs-based hydrogels at different $MgCl_2$ concentrations and RFs concentrations *versus* time scan mode and frequency scan mode.

Supplementary Fig. 4 | Rheological characterization of FFs-based hydrogels at different $MgCl_2$ concentrations and RFs concentrations *versus* time scan mode and frequency scan mode.

Supplementary Fig. 5 | UV-vis absorption curves of RFs, FFs, ICG, RFs-ICG, and FFs-ICG.

Supplementary Fig. 6 | Photothermal curves of FFs-ICG under different NIR power exposure and ICG concentrations.

Supplementary Fig. 7 | Photothermal curves of 5-day-old samples of FFs-ICG and pristine ICG.

Supplementary Fig. 8 | Behavior of FFs-ICG hydrogels exposed to NIR irradiation and cooling process.

Supplementary Fig. 9 | Temperature-dependent rheological changes of RFs-based hydrogels with different lysozyme fibril concentrations (0.5 and 1.5 wt.%) ranging from 20°C to 60°C.

Supplementary Fig. 10 | Temperature-dependent rheological changes of FFs-based hydrogels with different lysozyme fibril concentrations (0.5 and 1.5 wt.%) ranging from 20°C to 60°C.

Supplementary Fig. 11 | Representative ThT-staining images and the corresponding fluorescence intensity of FFs-ICG hydrogels before and after NIR irradiation for the analysis of photothermal-induced disassembling and reassembling behaviors.

Supplementary Fig. 12 | Representative calcein AM/PI-staining confocal images of L-929 cells for live/dead cell analysis after different treatments.

Supplementary Fig. 13 | Cell viability assays of FFs-ICG hydrogel with different FFs concentration using MTT method.

Supplementary Fig. 14 | Cytocompatibility assays of lysozyme monomers, FFs, RFs, and FFs-ICG hydrogel with different FFs concentration using HUVECs by MTT method.

Supplementary Fig. 15 | Hemolysis ratio assays of FFs-ICG hydrogel with different FFs concentrations, and different fibrils samples (RFs and FFs), and their corresponding tube images.

Supplementary Fig. 16 | The standard curve of lysozyme monomer concentration *vs.* absorbance.

Supplementary Fig. 17 | SEM images of MRSA and *P. aeruginosa* after treatment with FFs-ICG groups.

Supplementary Fig. 18 | Zeta potentials of different samples including ICG, RFs, FFs, RFs-ICG, FFs-ICG, MRSA and *P. aeruginosa*.

Supplementary Fig. 19 | Representative plate spreading images of MRSA strains after different treatments.

Supplementary Fig. 20 | Leaked NAG activity of MRSA strains after different treatments.

Supplementary Fig. 21 | Leaked K^+ concentrations of MRSA strains after different treatments.

Supplementary Fig. 22 | Antimicrobial activity of FFs-ICG against *P. aeruginosa* and *C. albicans* strains.

Supplementary Fig. 23 | MTT assay of L-929 cell proliferation cultured with supernatant from FFs-ICG treated macrophages.

Supplementary Fig. 24 | Wound area and traces in MRSA-infected murine wound model after various treatments.

Supplementary Fig. 25 | Comprehensive histological evaluation of healing conditions in MRSA-infected murine wound model after different treatments.

Supplementary Fig. 26 | Real-time quantitative PCR validation of mRNA expression of the genes related to key reparative cytokines and growth factors, including *Vegfa, Hif1α, Il10, and Pdgfb*, in wound tissues of MRSA-infected mice after FFs-ICG treatment.

Supplementary Fig. 27 | *In-vivo* therapeutic efficacy evaluation of FFs-ICG hydrogel in a MRSA-infected mice wound model with mature biofilm (3 days).

Supplementary Fig. 28 | *In-vivo* biosafety assessments in a MRSA-infected murine model.

Supplementary Fig. 29 | Hematological analysis of MRSA-infected mice on day 14 after various treatments.

Supplementary Fig. 30 | Analysis of platelet activation and thrombus formation after FFs-ICG treatment.

Supplementary Fig. 31 | *In-vivo* therapeutic efficacy evaluation of FFs-ICG hydrogel in non-infected murine wound model.

Supplementary Fig. 32 | *In-vivo* therapeutic efficacy evaluation of different hydrogels in a MRSA-infected prosthetic joint implants (PJI) murine model.

Supplementary Fig. 33 | *In-vivo* therapeutic efficacy evaluation of different hydrogels in a MRSA-infected porcine wound model.

Supplementary methods

Characterization

Thioflavin T (ThT) fluorescence spectroscopy was used to detect the content of beta-sheet structures in the process of amyloid fibrillation. The fluorescence assays were performed using a Hitachi FL-4600 fluorescence spectrometer. The fluorescence excitation wavelength was set at 438 nm, and the wavelength scanning range was 450-700 nm. The excitation and emission slit widths were set as 5 nm. All measurements were repeated three times. Circular dichroism (CD) was used to evaluate the changes in the secondary structure of flexible and rigid fibrils. The CD spectra of FFs and RFs were recorded using a CD spectrometer (Jasco J-815, Japan) with a quartz cuvette (optical length of 1 mm), a wavelength range of 195-250 nm, a bandwidth of 1 nm, and a scanning speed of 50 nm/min. All measurements were repeated three times, and the average values were plotted using Pro-Data Viewer software. Transmission electron microscopy (TEM) was performed using a JEM-2100 transmission electron microscope (TEM). The samples were stained with 2% phosphotungstic acid for 30 s and washed with ultrapure water before testing. Atomic force microscopy (AFM) was performed using a Bruker multimode 8 scanning probe microscope (Bruker, USA) and AFM imaging was performed in soft-tapping mode using a commercial silicon nitride cantilever (Bruker, USA) and a relatively soft tip-sample interaction. AFM samples were prepared by depositing an aliquot of diluted lysozyme solution at 0.1 mg/mL on a freshly cleaved mica for 2 min, followed by a gentle rinse and drying. AFM images were flattened using Nanoscope 8.1 software (Bruker, USA) and further analyzed using the FiberApp[1,2]. Scanning electron microscopy (SEM) was carried out using a Hitachi Field Emission Scanning Electron Microscope S-3400N from Japan. Ultraviolet-visible (UV-vis) curves were detected and recorded using a UV-Vis spectrophotometer (UV-5100 Japan Hitachi) with a scanning wavelength of 200-1100 nm. Zeta potential measurements were conducted using a nanoparticle size and zeta potential analyzer (ZetasizerNano ZS Malvern, UK).

Photothermal performance analysis

The photothermal performance of various samples (FFs, ICG, FFs-ICG) was characterized by Fotric X thermography (Fotric 285) under the irradiation of 808 nm laser according to previous studies[3,4]. To determine the optimal power, FFs-ICG were first formed hydrogel with 200 mM $MgCl_2$ solution at room temperature and then treated with 808 nm laser under different power densities (0.3-1 W/cm^2) for 10 min, respectively. To determine the optimal concentration, the FFs-ICG were diluted to different concentrations (0.1 mg/mL-0.3 mg/mL) with PBS buffer, and then irradiated with 808 nm laser of specific power density (0.5 W/cm^2) for 10 min, respectively. To compare the photothermal conversion capability, different types of samples (PBS, FFs, ICG, FFs-ICG) were diluted to 0.2 mg/mL with PBS buffer, and then irradiated with an 808 nm laser of a specific power density (0.5 W/cm^2) for 10 min, respectively. To further evaluate the photothermal stability and cycling, the temperature-time curves of FFs-ICG and pristine ICG molecules (0.2 mg/mL) were recorded by three cycles of laser (0.5 W/cm^2) on/off. All the above corresponding temperature-time curves and images were recorded using the software of Fotric 285.

Cytocompatibility characterization

The mouse fibroblasts (L-929) and human umbilical vein endothelial cells (HUVECs) were used to verify the cytocompatibility of as-prepared samples. Specifically, 1×10^4 cells/well were cultured in DMEM medium including 10% fetal bovine serum (FBS) and 1% penicillin/streptomycin in 96-

well plates at 37°C in a humid atmosphere of 5% CO_2. Different concentrations of FFs hydrogels and different kinds of other samples (Lys, RFs and FFs, with a final concentration of 100 μg/mL) were added to the above cells with replaced FBS-free medium and incubated for 24 hours, respectively. Then the previous medium was discarded, and 100 μL of fresh DMEM containing 5 mg/mL MTT was added to each well and incubated at 37 °C for another 2 hours. Then, 100 μL was pipetted to a brand-new 96-well plate, and the absorbance was measured by a microplate reader (Spark Tecan, Switzerland) at 490 nm. For CLSM experiments, the cells were firstly washed with sterile PBS (10 mM, pH=7.4) for three times. Dye mixtures (100 μL) of calcein acetoxymethyl ester (calcein-AM) and propidium iodide (PI) were added to each well and further incubated for 30 min in the dark environment. Then, the cells were observed and recorded by CLSM. At least 3 replicate samples were measured in each group.

Hemocompatibility characterization

One milliliter of fresh blood was collected from the venous plexus behind the eye sockets of mice, and immediately added to the anticoagulation tube for assessing the hemocompatibility. Acquired fresh blood was centrifuged at 2500 rpm/min for 15 min at 4°C. Erythrocytes were collected and resuspended in 10 mL of PBS buffer and washed five more times with PBS buffer. These erythrocytes were resuspended in 1 mL of PBS buffer and divided into 200 μL per tube. Different concentrations of FFs hydrogels and different kinds of other samples (Lys, RFs and FFs, with a final concentration of 100 μg/mL) were added to the above prepared tubes, and the tubes were incubated at room temperature for 4 hours. PBS buffer was used as a negative control, and TritonX-100 was used as a positive control. Next, these tubes were centrifuged at 10,000 rpm/min for 10 min, and then the upper layer of the solution was taken and transferred to another new 96-well plate. The absorbance was measured at 541 nm with a microplate reader (Spark Tecan, Switzerland), and at least three replicate samples were measured for each group.

***In-vitro* antimicrobial activity assays**

Gram-positive bacteria methicillin-resistant *Staphylococcus aureus* (MRSA), Gram-negative bacteria *Pseudomonas aeruginosa*, and a fungal strain *Candida albicans* were selected for the evaluation of *in-vitro* antibacterial efficacy according to previous studies[5,6]. Prior to this experiment, the bacteria were cultured on LB agar plates and incubated at 37°C overnight. A single colony was collected and incubated in 4 mL of liquid LB medium at 37°C overnight. After centrifugal washing of the above bacteria with sterile PBS (10 mM, pH 7.4) for three times, the bacteria were diluted to 10^6 CFU/mL for the subsequent experiments. Firstly, the samples (1 mL) in each group (PBS, Lys, RFs, FFs, ICG, RFs-ICG, FFs-ICG) were prepared in the bottom of 24-well plates, and then 500 μL of the above bacterial suspensions (1×10^6 CFU/mL) was added to each well and incubated at 37°C for 8 hours. CFU counting method was used to evaluate the antibacterial efficacy. The survival status of MRSA after different treatments were characterized using a confocal laser scanning microscope (Zeiss, FV3000). According to the procedure of Live/Dead BacLight Bacterial Survival Kit (L13152, Invitrogen), these treated bacterial suspensions were gently rinsed three times with PBS, and then stained with 500 μL of mixed staining reagent (SYTO9 and PI dyes) under dark conditions for 30 min. Finally, the bacteria were observed by CLSM. Here, green fluorescence represents live bacteria, while red fluorescence represents dead bacteria.

Macrophage polarization assays

Mouse-derived macrophage (Raw246.7) was utilized to test magnesium ions (Mg^{2+})-mediated macrophage polarization towards M2 phenotype according to previous studies[7,8]. Specifically, 1×10^5 cells/well were cultured in DMEM medium including 10% fetal bovine serum (FBS) and 1% penicillin/streptomycin in 96-well plates at 37°C in a humidified atmosphere of 5% CO_2. After 12-hour incubations, the medium in cell cultures was replaced by the FBS-free medium. Meanwhile, different samples (PBS, IL-4, RFs, FFs, RFs-ICG, and FFs-ICG) were prepared as hydrogel, then immersed in 1 mL of FBS-free medium. RFs-ICG and FFs-ICG groups were treated using 808 nm laser irradiation for 10 min. Subsequently, these samples were filtered by using a 0.22 μm filter membrane, and then the Mg^{2+}-containing extract solution was added to the above cell cultures and incubated for 1 day. The cells were collected by discarding the old medium, and each well was fixed by adding 80% methanol solution for 40 min at 4°C. After centrifugation, the excess solution was removed, and the fresh DMEM containing 5 μL of CD206 antibody with red fluorescence was added and incubated at 37°C for 60 min. Next, the superfluous unbinding dyes were removed prior to macrophage polarization analysis using flow cytometry. For CLSM imaging, the treated cells were first fixed with the addition of 80% methanol solution at 4°C for 30 min, after which the excess solution was removed and fresh DMEM containing 5 μL of CD206 antibody with red fluorescence was added to stain the samples for 60 min. After the excess dye was removed, the polarization status of cells was observed and recorded using CLSM. At least 3 replicate samples were measured in each group.

***In-vivo* platelet activation and thrombus formation assays**

To evaluate the ability of FFs-ICG to induce platelet activation and thrombus formation, blood samples were collected from non-infected mice on day 10 after treatment with FFs-ICG. Blood from PBS group served as the negative control, while thrombin was added to the blood collected from the PBS group as the positive control. Subsequently, single-cell suspensions were stained with fluorescence-labeled mouse flow cytometry antibodies (CD61 and CD62P) and analyzed using a flow cytometer (Beckton Dickinson, USA). Additionally, 100 μL of blood samples from each group was pipetted into a brand-new 96-well plate, and the absorbance was measured at 540 nm using a microplate reader (Spark Tecan, Switzerland). The blood coagulation index was calculated using the following formula:

$$\text{BCI (\%)} = \frac{\text{ODsample} \times \text{ODpositive}}{\text{ODnegative}} \times 100\%$$

***In-vivo* qPCR analysis of murine wound tissues**

On day 18 treatment, skin tissues (0.5 cm×0.5 cm) were collected from the wound of MRSA-infected mice treated with FFs-ICG or PBS control. Single-cell suspensions were prepared through a 70-μm cell strainer (Beyotime Biotechnology, China). RNA extraction was performed from the single-cell suspensions of each group using the *SteadyPure* Quick RNA Extraction Kit (Accurate Biotechnology, China) according to the manufacturer's instructions. The extracted RNA was then reverse transcribed into cDNA using the *Evo* M-MLV RT Mix Kit (Accurate Biotechnology, China). Real-time quantitative PCR was used SYBR Green Premix Pro Taq HS qPCR Kit (Accurate Biotechnology, China) to perform the expression levels of target genes. The amplification protocol consisted of an initial denaturation at 95°C for 5 min, followed by 40 cycles of 95°C for 10 s and

60°C for 30 s. Sequence-specific primers used are listed in **Table S1**. The GAPDH gene was used as an internal reference, and relative RNA levels of each target gene were calculated using the 2^(-*ΔΔCt*) method.

Effect of polarized macrophages on L-929 cell proliferation

Consistent with the aforementioned methodologies for Cytocompatibility characterization and Macrophage polarization assays, L-929 and Raw 264.7 cells were cultured in 96-well plates. Simultaneously, FFs-ICG was prepared as hydrogel, then add 1 mL in FBS-free medium. Subsequently, it was treated using 808 nm laser irradiation for 10 min. Then it was filtered by using a 0.22 μm filter membrane, and then the Mg^{2+}-containing leaching solution was added to Raw 264.7 cells and incubated for 24 h. The supernatant from the Raw 264.7 culture medium was then collected and mixed at a 1:1 ratio with DMEM medium including 10% fetal bovine serum (FBS) and 1% penicillin/streptomycin. This mixed medium was added to L-929 cells at 200 μL per well and incubated for 24 h. Finally, following the identical MTT method described in the Cytocompatibility characterization section, the absorbance was measured by a microplate reader (Spark Tecan, Switzerland) at 490 nm.

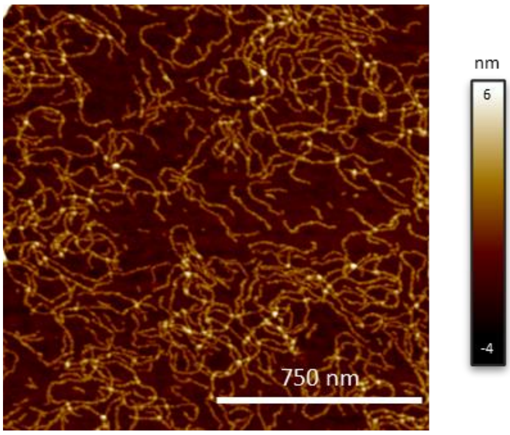

Supplementary Fig. 1 | AFM images of flexible fibrils (FFs) that were diluted to 0.002 mg/mL before deposition on mica. (Scale bar: 750 nm)

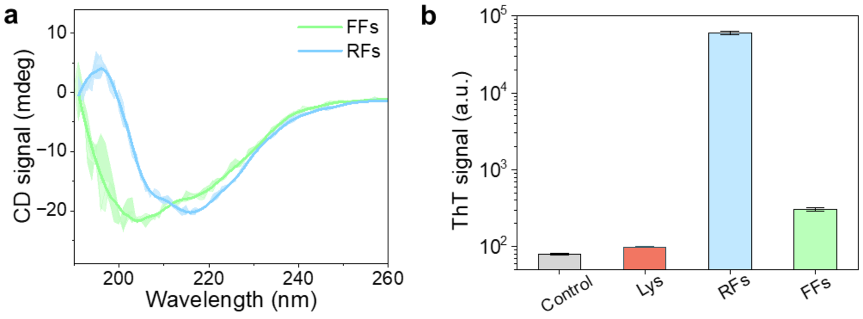

Supplementary Fig. 2 | Secondary structural characterization of RFs and FFs. **a-b,** CD spectra (**a**) and ThT fluorescence intensity assays (**b**) of RFs and FFs. PBS solution and lysozyme monomer solution are measured as the controls in the ThT fluorescence intensity assays (n=3). Source data are provided as a Source Data file.

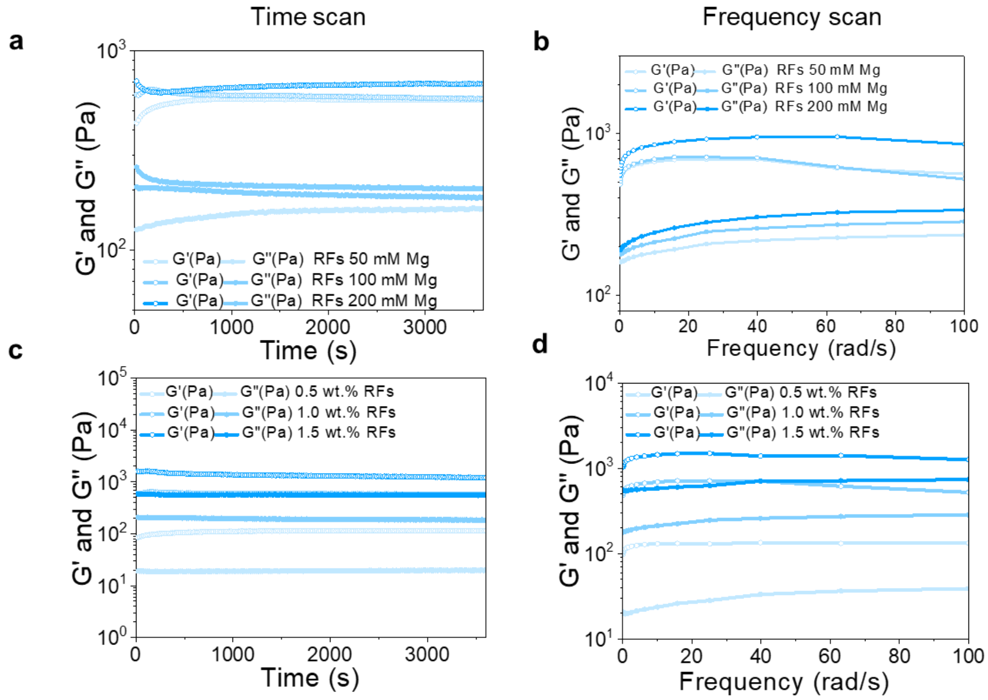

Supplementary Fig. 3 | Rheological characterization of RFs-based hydrogels. **a-b,** Rheological time scan mode characterization of RFs-based hydrogels at different $MgCl_2$ concentrations (**a**) and RFs concentrations (**b**). **c-d**, Rheological frequency scan mode characterization of RFs-based hydrogel at different $MgCl_2$ concentrations (**c**) and RFs concentrations (**d**). Source data are provided as a Source Data file.

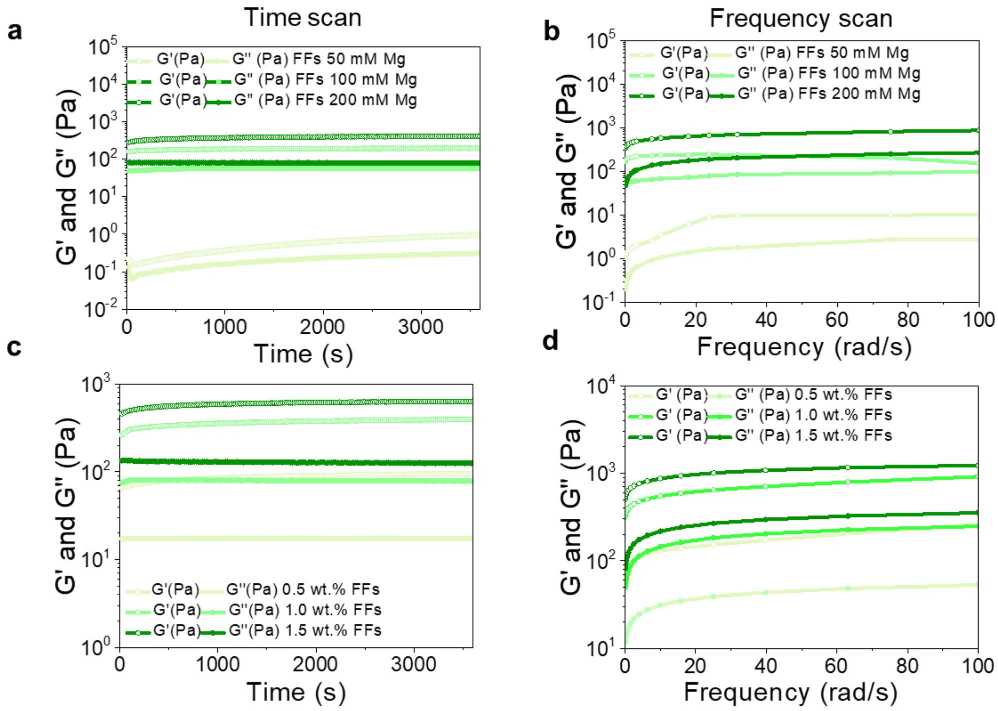

Supplementary Fig. 4 | Rheological characterization of FFs-based hydrogels. **a-b,** Rheological time scan mode characterization of FFs-based hydrogel at different $MgCl_2$ concentrations (**a**) and FFs concentrations (**b**). **c-d**, Rheological frequency scan mode characterization of FFs-based hydrogel at different $MgCl_2$ concentrations (**c**) and FFs concentrations (**d**). Source data are provided as a Source Data file.

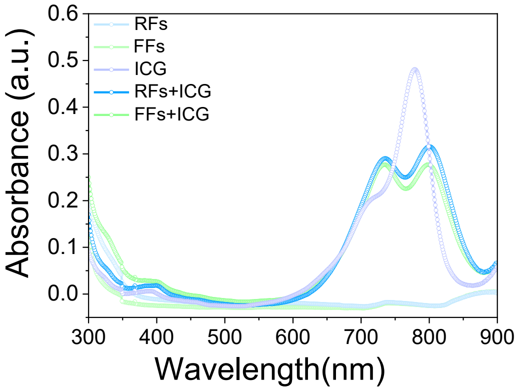

Supplementary Fig. 5 | UV-vis absorption curves of RFs, FFs, ICG, RFs-ICG, and FFs-ICG. Source data are provided as a Source Data file.

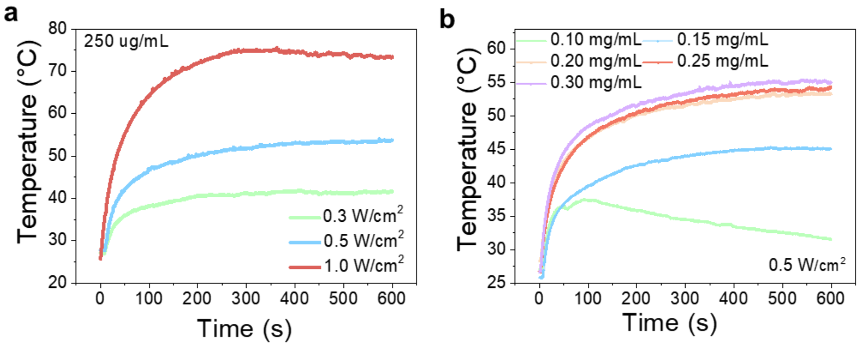

Supplementary Fig. 6 | Photothermal performance of FFs-ICG nanonets. **a**, Photothermal curves of FFs-ICG (250 μg/mL) exposed to NIR with different power intensities. **b**, Photothermal curves of FFs-ICG (0.5 W/cm^2) at different ICG concentrations.

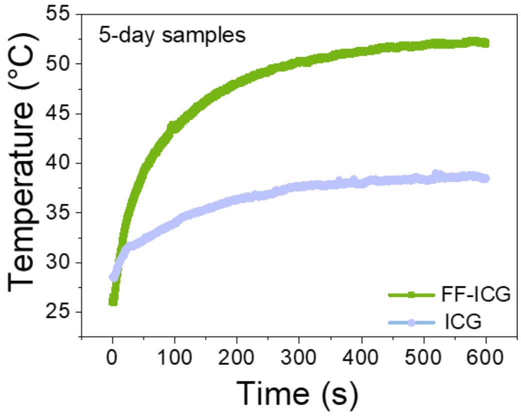

Supplementary Fig. 7 | Photothermal curves of 5-day-old samples of FFs-ICG and pristine ICG. Source data are provided as a Source Data file.

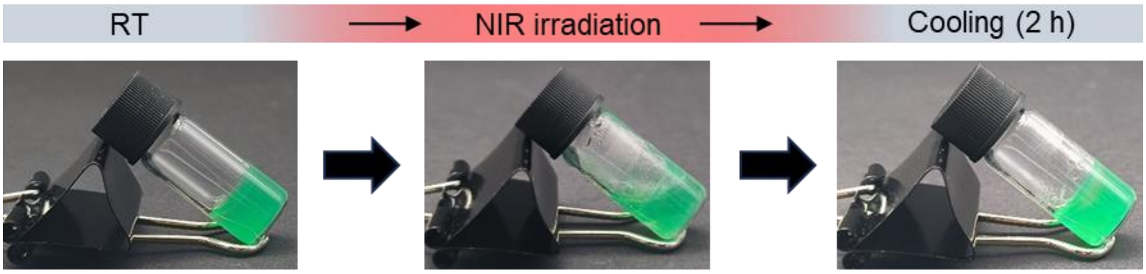

Supplementary Fig. 8 | Behavior of FFs-ICG hydrogel images upon NIR exposure followed by subsequent cooling process.

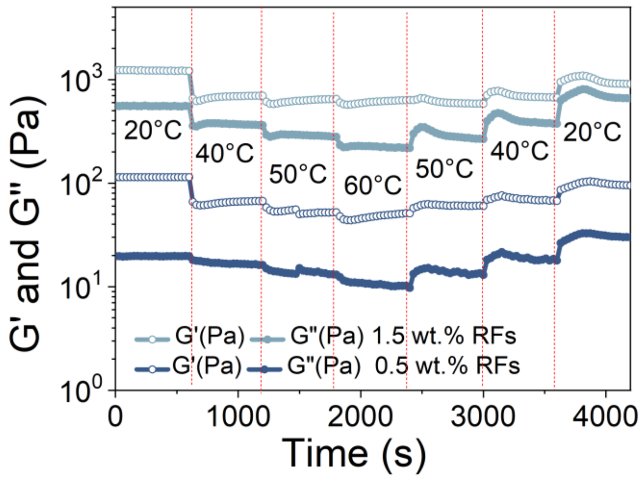

Supplementary Fig. 9 | Temperature-dependent rheological changes of RFs-based hydrogels with different lysozyme fibril concentrations (0.5 and 1.5 wt.%) ranging from 20ºC to 60ºC. Source data are provided as a Source Data file.

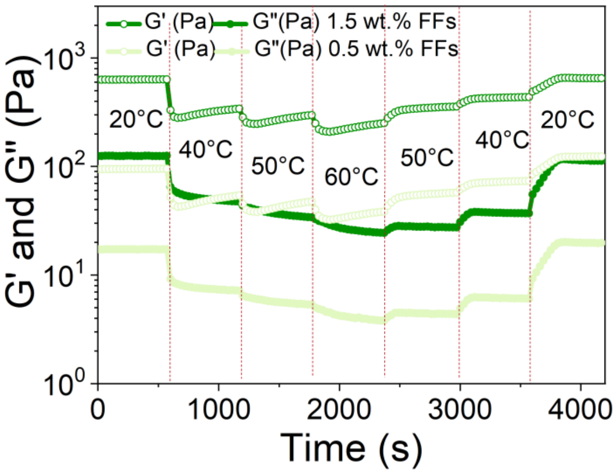

Supplementary Fig. 10 | Temperature-dependent rheological changes of FFs-based hydrogels with different lysozyme fibril concentrations (0.5 and 1.5 wt.%) ranging from 20°C to 60°C. Source data are provided as a Source Data file.

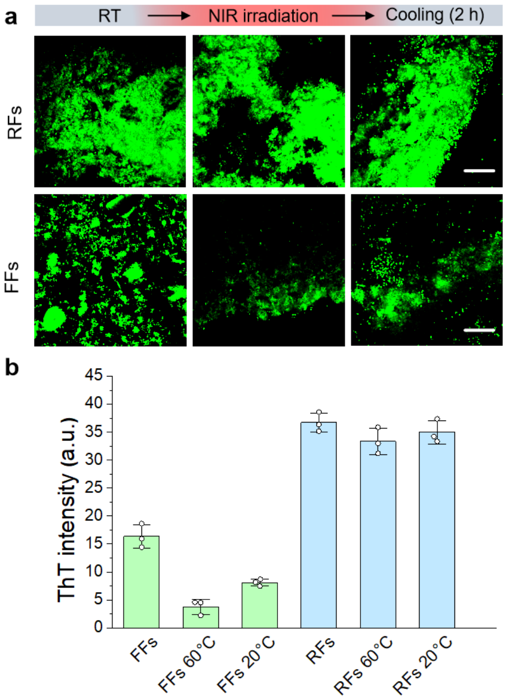

Supplementary Fig. 11 | Photothermal-induced ThT fluorescence intensity changes of FFs-ICG hydrogels. **a-b**, Representative ThT-staining images (scale bar: 200 μm) (**a**) and the corresponding fluorescence intensity statistics (**b**) (n = 3; data are presented as individual points) of FFs-ICG hydrogel before and after NIR irradiation for the analysis of photothermal-induced disassembling and reassembling behaviors. Source data are provided as a Source Data file.

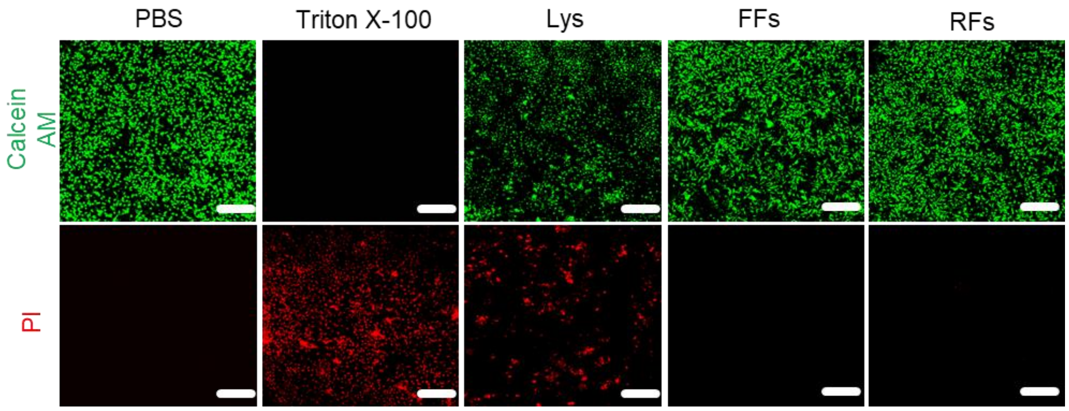

Supplementary Fig. 12 | The representative Calcein acetyl methoxymethyl ester (Calcein-AM)/propidium iodide (PI)-staining confocal images of L-929 cells for live/dead cell analysis after different treatments. Scale bar: 200 μm. Both FFs and RFs hydrogels exhibit high cell viability indicating that the linear alignment lysozyme limits the cytotoxicity of native lysozyme.

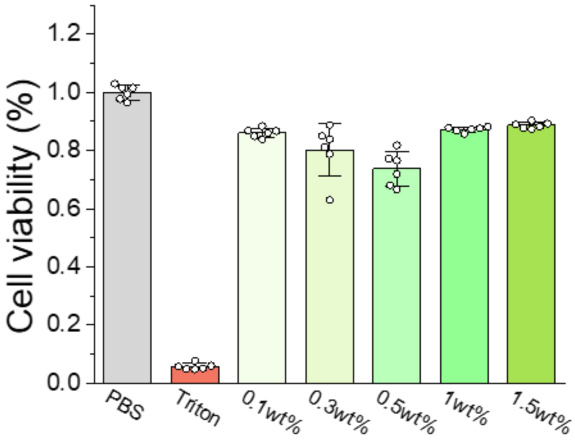

Supplementary Fig. 13 | Cell viability assays of FFs-ICG hydrogel with different FFs concentration using MTT method (n = 6; data are presented as individual points). PBS solution and 0.1% Triton X-100 are set as the controls. Source data are provided as a Source Data file.

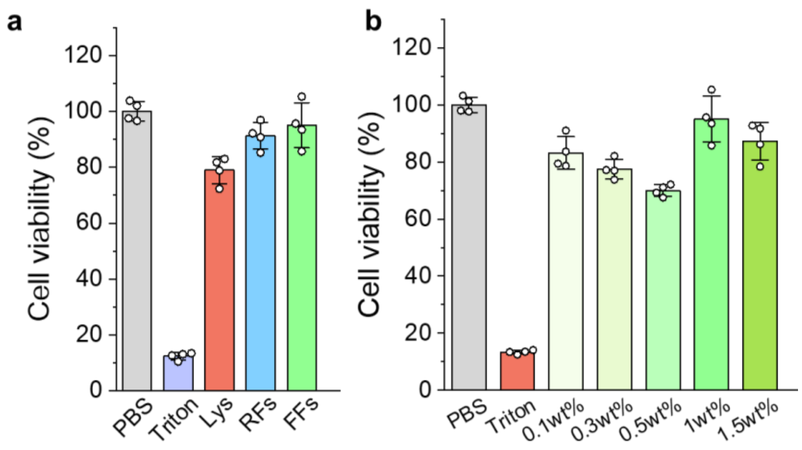

Supplementary Fig. 14 | Cytocompatibility assays against HUVECs by MTT method. **a,** Cytocompatibility assays of lysozyme monomers, FFs, and RFs at concentration of 1.0 wt.% using MTT method in HUVEC cells (n = 4; data are presented as individual points). **b,** Cytocompatibility assays of FFs-ICG hydrogel with different FFs concentration using MTT method in HUVEC cells (n = 4; data are presented as individual points). PBS solution and 0.1% Triton X-100 are set as the controls. Source data are provided as a Source Data file.

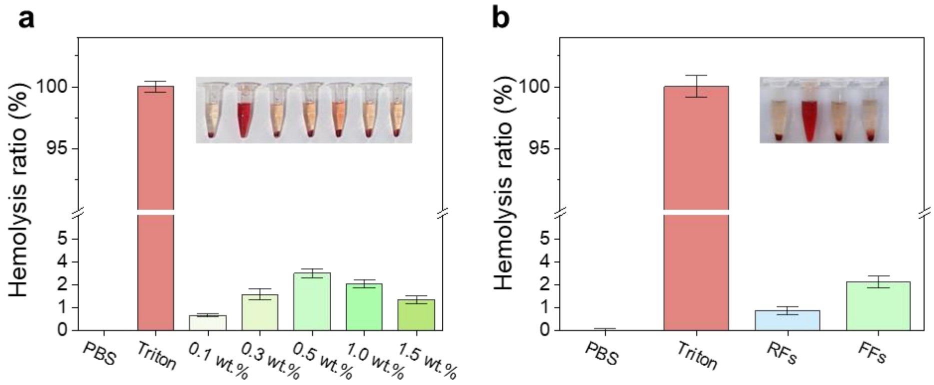

Supplementary Fig. 15 | Hemocompatibility assays. **a**, Hemolysis ratio assays (n = 3) of FFs-ICG hydrogel with different FFs concentrations, and their corresponding tube images. **b,** Hemolysis ratio (n = 3) of RFs and FFs (1.0 wt.%), and their corresponding tube images. PBS solution and 0.1% Triton X-100 are set as the controls. Source data are provided as a Source Data file.

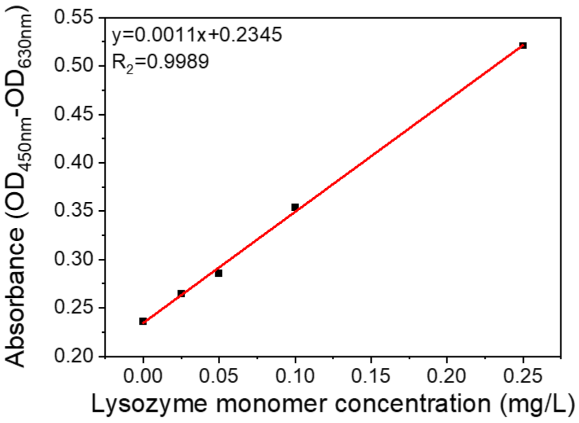

Supplementary Fig. 16 | The standard curve of absorbance *vs.* lysozyme monomer concentration. Source data are provided as a Source Data file.

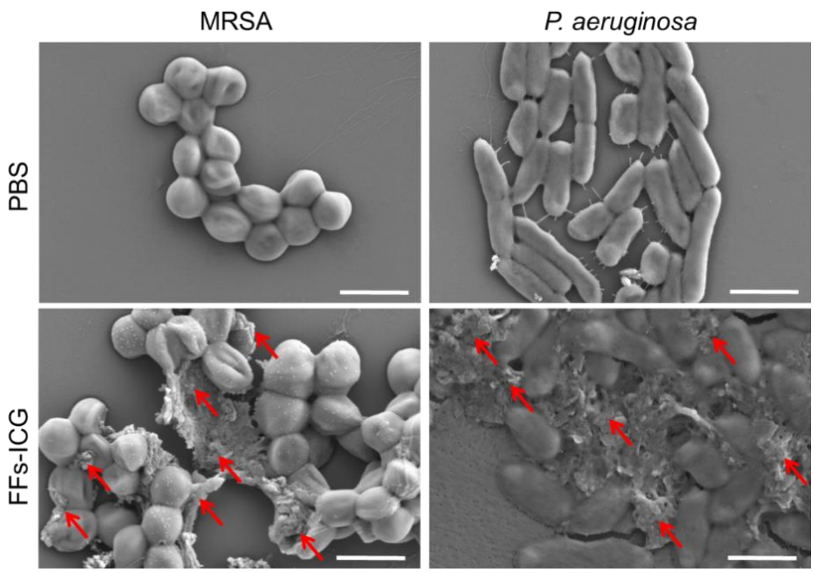

Supplementary Fig. 17 | The SEM images of MRSA and *P. aeruginosa* after treatment with FFs-ICG groups. Scale bar: 1 μm. Note: red arrows represent the FFs networks.

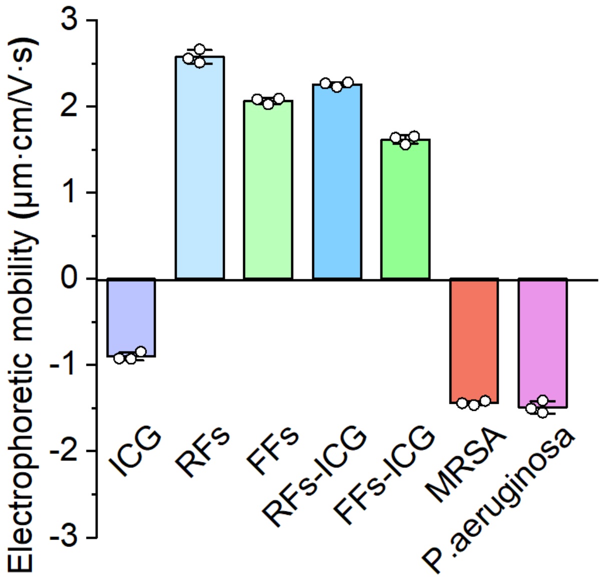

Supplementary Fig. 18 | Zeta potentials (n = 3; data are presented as individual points) of different samples including ICG, RFs, FFs, RFs-ICG, FFs-ICG, MRSA and *P. aeruginosa*. Source data are provided as a Source Data file.

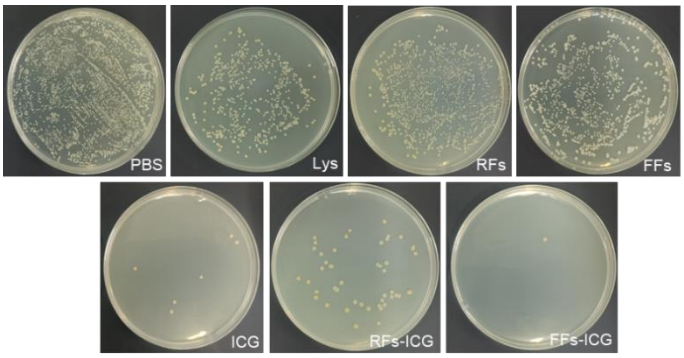

Supplementary Fig. 19 | Representative plate spreading images of MRSA strains after different treatments.

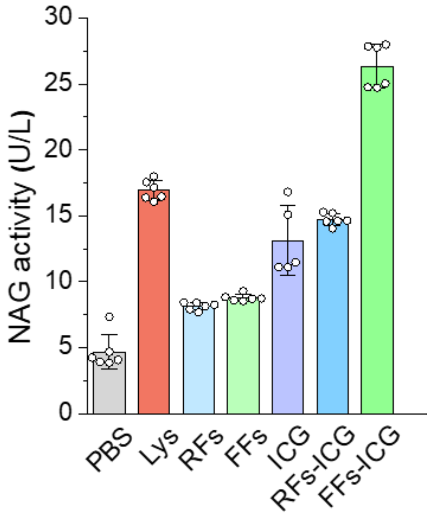

Supplementary Fig. 20 | Leaked NAG activity (n = 6; data are presented as individual points) of MRSA strains after different treatments. Source data are provided as a Source Data file.

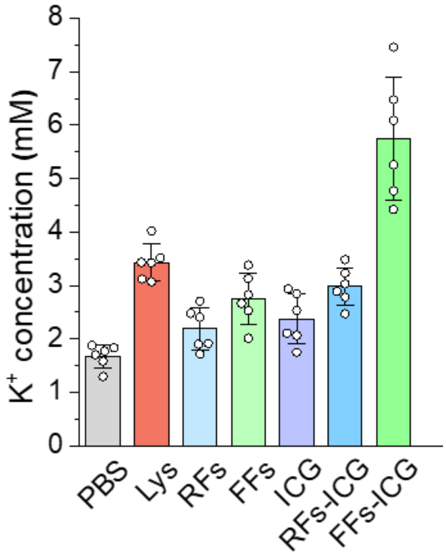

Supplementary Fig. 21 | Leaked K^+ concentrations (n = 6; data are presented as individual points) of MRSA strains after different treatments. Source data are provided as a Source Data file.

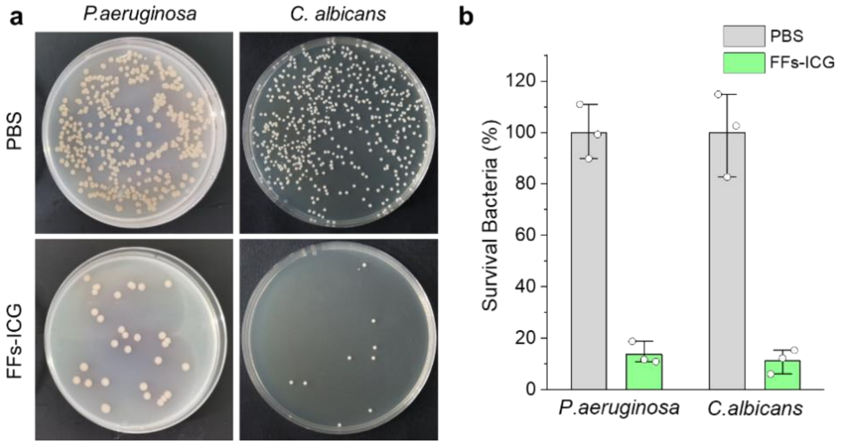

Supplementary Fig. 22 | Antimicrobial activity of FFs-ICG against *P. aeruginosa* and *C. albicans*. **a,** Representative plate spreading images of *P. aeruginosa* and *C. albicans* strains after treatment with FFs-ICG. **b,** Antibacterial efficiency assays (*P. aeruginosa* and *C. albicans*) of FFs-ICG using CFU counting methods (n = 3; data are presented as individual points). Source data are provided as a Source Data file.

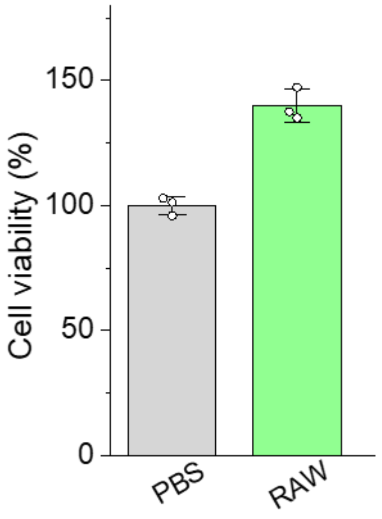

Supplementary Fig. 23 | MTT assay of L-929 cell proliferation cultured with the supernatant from FFs-ICG treated macrophages (n = 3; data are presented as individual points). Source data are provided as a Source Data file.

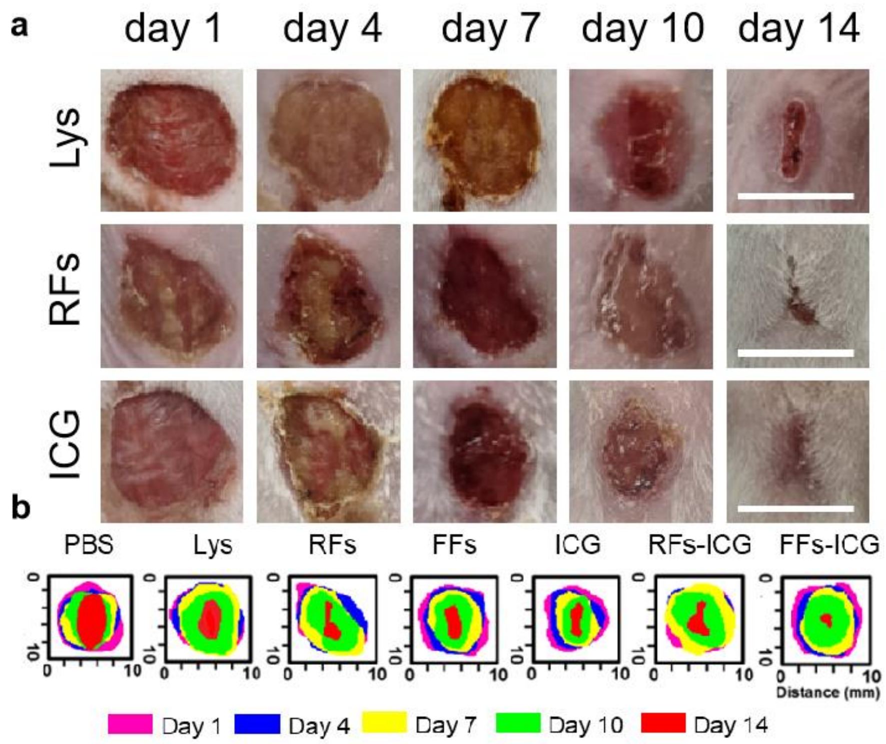

Supplementary Fig. 24 | Wound healing evaluation in a MRSA-infected murine wound model. **a,** Representative macroscopic wound images of MRSA-infected mice after various treatments, including lysozyme monomer (Lys), RFs and ICG (Scale bar: 1 cm). **b,** Wound trace images of MRSA-infected mice after various treatments.

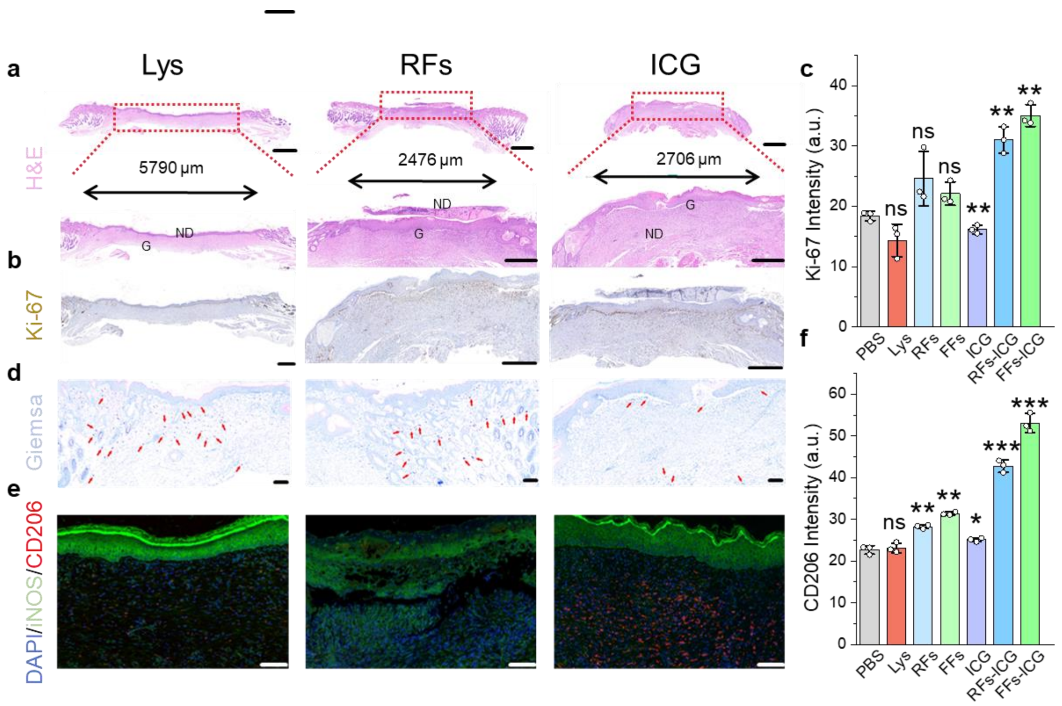

Supplementary Fig. 25 | Comprehensive histological evaluation of healing conditions in MRSA-infected murine wound model after different treatments. **a,** Representative H&E staining images of wounds for the analysis of dermis and epidermis regeneration in different treatment groups on day 14. Scale bar: 2 mm (top) and 500 μm (down). **b,** Representative Ki-67-staining images of wounds for the analysis of cell proliferation in different treatment groups on day 14. Scale bar: 500 μm. **c,** Quantitative analysis of Ki-67 intensity (n = 3; data are presented as individual points) in wound tissues from different treatment groups on day 14 (Lys (p = 0.0841), RFs (p = 0.0900), FFs (p = 0.0700), ICG (p = 0.0054), RFs-ICG (p = 0.0085), FFs-ICG (p = 0.0039) *vs.* PBS). **d,** Representative Giemsa-staining images of wounds for the analysis of survival bacteria in different treatment groups on day 14 (the red arrows represent the residual bacteria). Scale bar: 500 μm. **e,** The representative iNOS and CD206-stainin images of wounds for the analysis of macrophage polarization in different treatment groups on day 14. Scale bar: 100 μm. **f,** Quantitative analysis of CD206 intensity (n = 3; data are presented as individual points) in wound tissues from different treatment groups on day 14 (Lys (p = 0.2979), RFs (p = 0.0078), FFs (p = 0.0020), ICG (p = 0.0475), RFs-ICG (p = 0.0002), FFs-ICG (p = 0.0005) *vs.* PBS). The data were expressed as mean ± standard deviation (S.D). Statistical significance was analyzed by one-way ANOVA using GraphPad Prism 8, followed by Tukey's post-hoc test for pairwise comparisons. Statistical significance was defined as * $p < 0.05$, ** $p < 0.01$, and *** $p < 0.001$ *vs.* PBS. Source data are provided as a Source Data file.

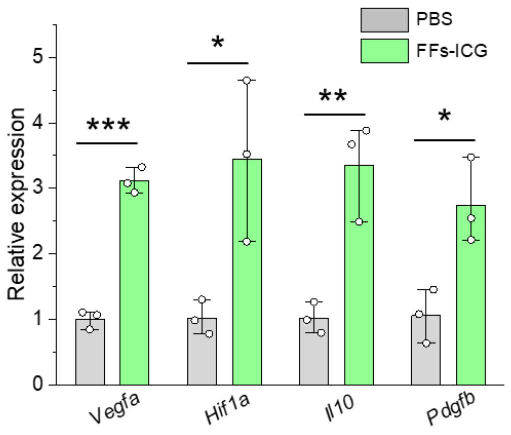

Supplementary Fig. 26 | Real-time quantitative PCR validation of mRNA expression of the genes related to key reparative cytokines and growth factors, including *Vegfa* (FFs-ICG (p = 0.0008) *vs.* PBS), *Hif1α* (FFs-ICG (p = 0.0446) *vs.* PBS), *Il10* (FFs-ICG (p = 0.0091) *vs.* PBS), and *Pdgfb* (FFs-ICG (p = 0.0368) *vs.* PBS), in wound tissues of MRSA-infected mice after FFs-ICG treatment (n = 3; data are presented as individual points). Statistical significance was defined as * $p < 0.05$, ** $p < 0.01$, and *** $p < 0.001$ *vs.* PBS. Source data are provided as a Source Data file.

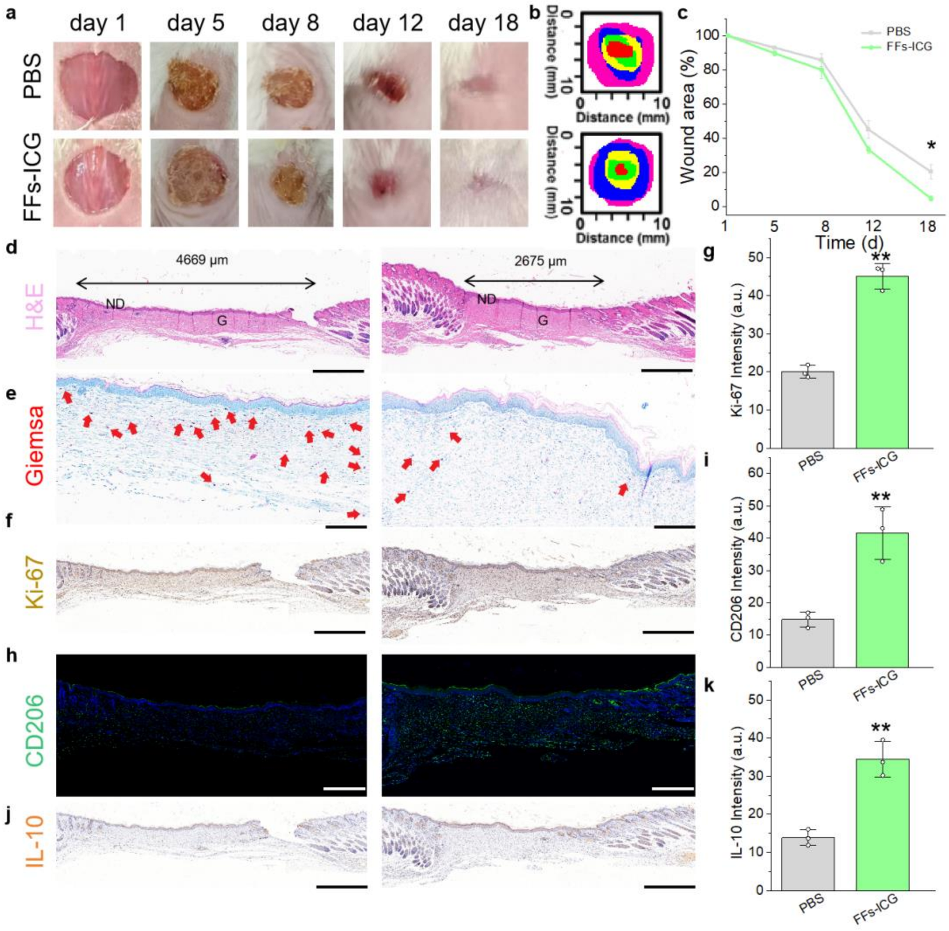

Supplementary Fig. 27 | *In-vivo* therapeutic efficacy evaluation of FFs-ICG hydrogel in a MRSA-infected mice wound model with mature biofilm (3 days). **a,** The representative macroscopic wound images of MRSA-infected mice after FFs-ICG treatments (Scale bar: 1 cm). **b,** Wound trace images of MRSA-infected mice after FFs-ICG treatments. **c,** Wound area quantification of MRSA-infected mice after FFs-ICG treatments (n = 3; FFs-ICG (p = 0.0157) *vs.* PBS). **d,** The representative H&E staining images of wounds for the analysis of dermis and epidermis regeneration in FFs-ICG treatment on day 14. Scale bar: 1 mm. **e,** Representative Giemsa-staining images of wounds for the analysis of survival bacteria in FFs-ICG treatment on day 14 (the red arrows represent the residual bacteria). Scale bar: 200 µm. **f,** The representative Ki-67-staining images of wounds for the analysis of cell proliferation in FFs-ICG treatment on day 14. Scale bar: 1 mm. **g,** Quantitative analysis of Ki-67 intensity (n = 3; data are presented as individual points; FFs-ICG (p = 0.0030) *vs.* PBS). **h,** The representative CD206-stainin images of wounds for the analysis of macrophage polarization in FFs-ICG treatment on day 14. Scale bar: 500 µm. **i,** Quantitative analysis of CD206 intensity (n = 3; data are presented as individual points; FFs-ICG (p = 0.0079) *vs.* PBS). **j,** The representative IL-10-staining images of wounds for the analysis of cell inflammation in FFs-ICG treatment on day 14. Scale bar: 1 mm. **k,** Quantitative analysis of IL-10 intensity (n = 3; data are presented as individual points; FFs-ICG (p = 0.0075) *vs.* PBS). The data were expressed as mean ± standard deviation (S.D). Statistical significance was analyzed by one-way ANOVA using GraphPad Prism 8, followed by Tukey's post-hoc test for pairwise comparisons. Statistical significance was defined as * $p < 0.05$, ** $p < 0.01$, and ***$p < 0.001$ *vs.* PBS. Source data are provided as a Source Data file.

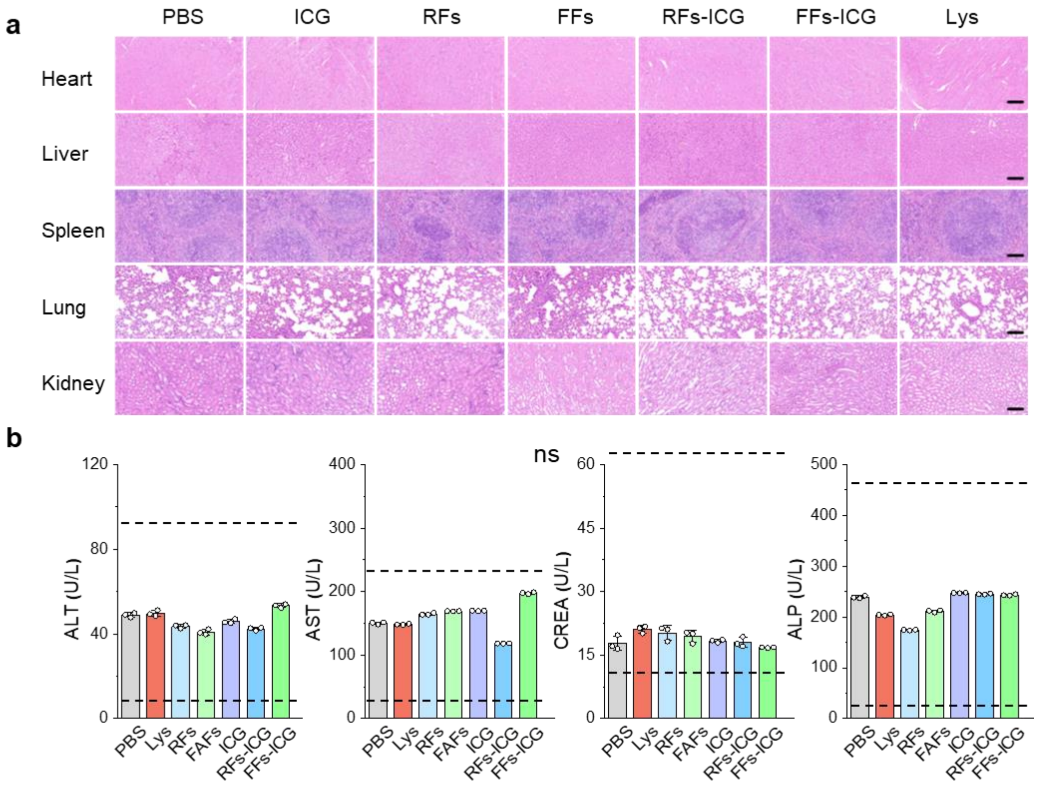

Supplementary Fig. 28 | *In-vivo* biosafety assessments in a MRSA-infected murine model. **a,** The representative H&E staining images of major organs (heart, liver, spleen, lung, and kidney) from MRSA-infected mice for the *in-vivo* biosafety characterization in different treatment groups on day 14. Scale bar: 100 μm. **b,** Blood biochemistry analysis, including creatinine (CREA), alkaline phosphatase (ALP), alanine aminotransferase (ALT), and aspartate aminotransferase (AST) of MRSA-infected mice on day 14 after the various treatments (n = 3; data are presented as individual points). Source data are provided as a Source Data file.

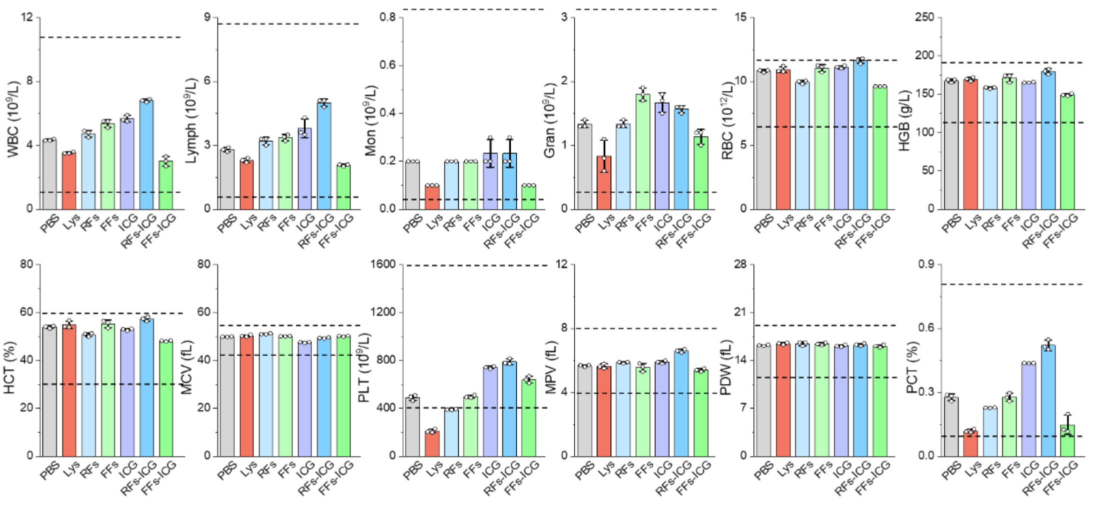

Supplementary Fig. 29 | Hematological analysis (WBC, Lymph, Mon, Gran, RBC, HGB, HCT, MCV, PLT, MPV, PDW and PCT) of MRSA-infected mice on day 14 after the various treatments (n = 3; data are presented as individual points). Note: WBC, total white blood cell count; Lymph, lymphocyte count; Mon, monocyte count; Gran, neutrophil granulocyte count; RBC, red blood cell counts; HGB, hemoglobin levels; HCT, hematocrit; MCV, mean corpuscular volume; PLT, platelet count; MPV, mean platelet volume; PDW, platelet distribution width; PCT, plateletcrit. The dotted lines indicate the upper and lower limits of the normal range. Source data are provided as a Source Data file.

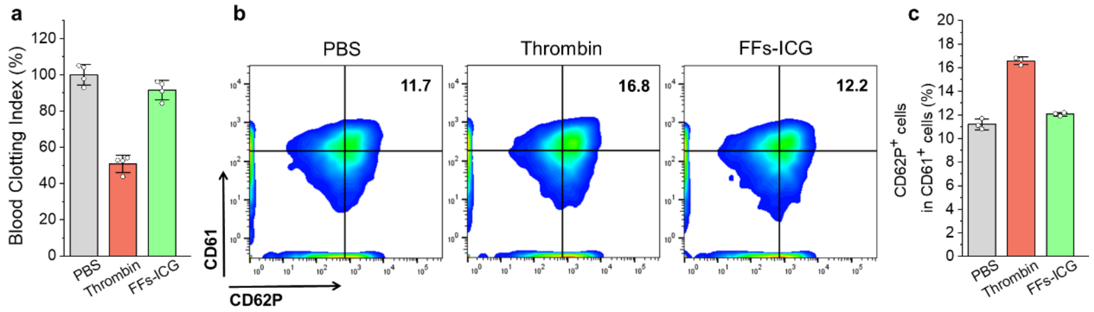

Supplementary Fig. 30 | Analysis of platelet activation and thrombus formation in blood after FFs-ICG treatment. **a,** Blood coagulation index (BCI) of PBS, Thrombin and FFs-ICG-treated blood from mice (n = 3; data are presented as individual points). **b,** Representative flow cytometry profiles of CD61^{+}CD62P^{+} cells, and **c,** the corresponding quantification of CD61^{+}CD62P^{+} cells (n = 3; data are presented as individual points) in the blood of mice treated with FFs-ICG. Source data are provided as a Source Data file.

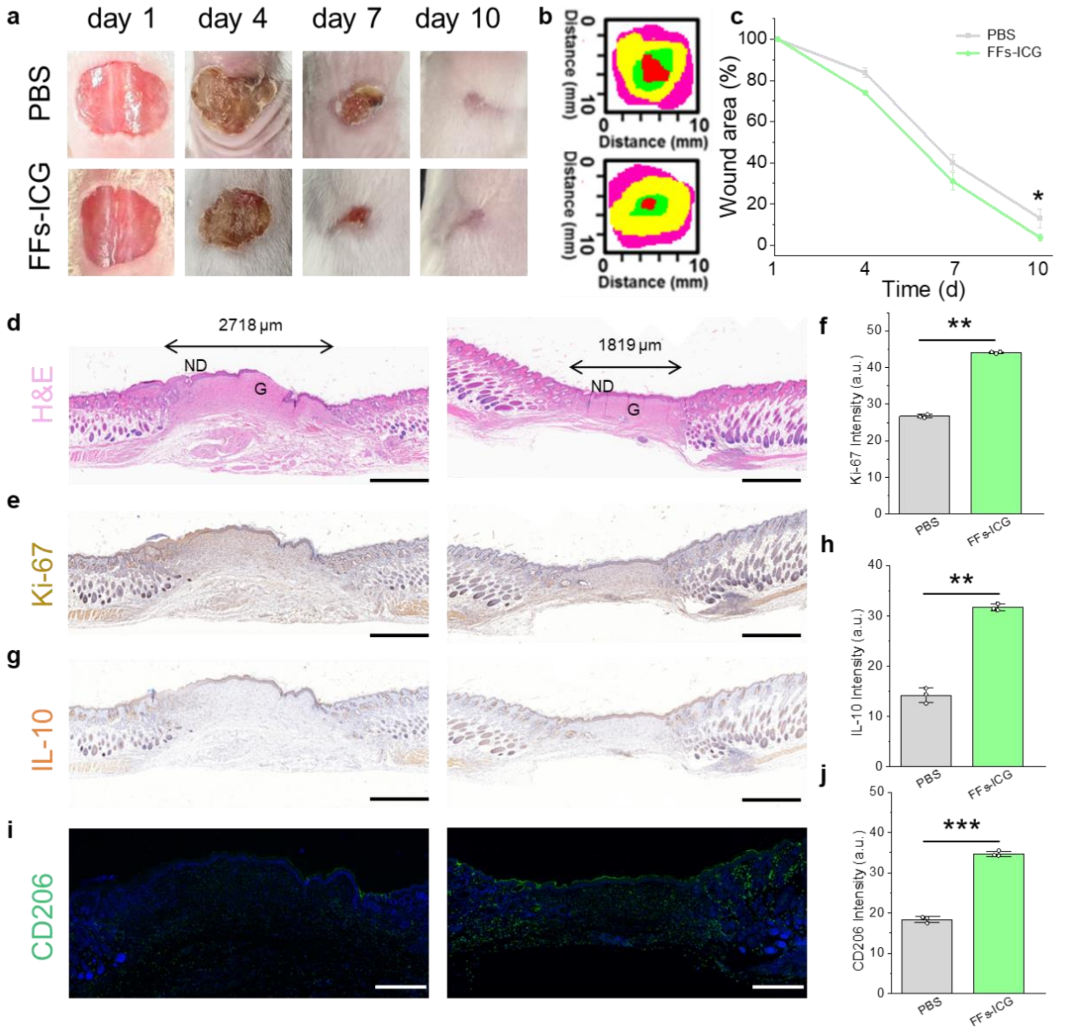

Supplementary Fig. 31 | *In-vivo* therapeutic efficacy evaluation of FFs-ICG hydrogel in non-infected murine wound model. **a,** The representative macroscopic wound images of healthy mice after FFs-ICG treatments (Scale bar: 1 cm). **b,** Wound trace images of healthy mice after FFs-ICG treatments. **c,** Wound area quantification of healthy mice after FFs-ICG treatments (n = 3; FFs-ICG (p = 0.0498) *vs.* PBS). **d,** The representative H&E staining images of wounds for the analysis of dermis and epidermis regeneration in FFs-ICG treatment on day 10. Scale bar: 1 mm. **e,** The representative Ki-67-staining images of wounds for the analysis of cell proliferation in FFs-ICG treatment on day 10. Scale bar: 1 mm. **f,** Quantitative analysis of Ki-67 intensity (n = 3; data are presented as individual points; FFs-ICG ($p < 0.0001$) *vs.* PBS). **g,** The representative CD206-stainin images of wounds for the analysis of macrophage polarization in FFs-ICG treatment on day 10. Scale bar: 500 μm. **h,** Quantitative analysis of CD206 intensity (n = 3; data are presented as individual points; FFs-ICG (p = 0.0024) *vs.* PBS). **i,** The representative IL-10-staining images of wounds for the analysis of cell inflammation in FFs-ICG treatment on day 10. Scale bar: 1 mm. **j,** Quantitative analysis of IL-10 intensity (n = 3; data are presented as individual points; FFs-ICG (p = 0.0013) *vs.* PBS). The data were expressed as mean ± standard deviation (S.D). Statistical significance was analyzed by one-way ANOVA using GraphPad Prism 8, followed by Tukey's post-hoc test for pairwise comparisons. Statistical significance was defined as *$p < 0.05$, **$p < 0.01$, and ***$p < 0.001$ *vs.* PBS. Source data are provided as a Source Data file.

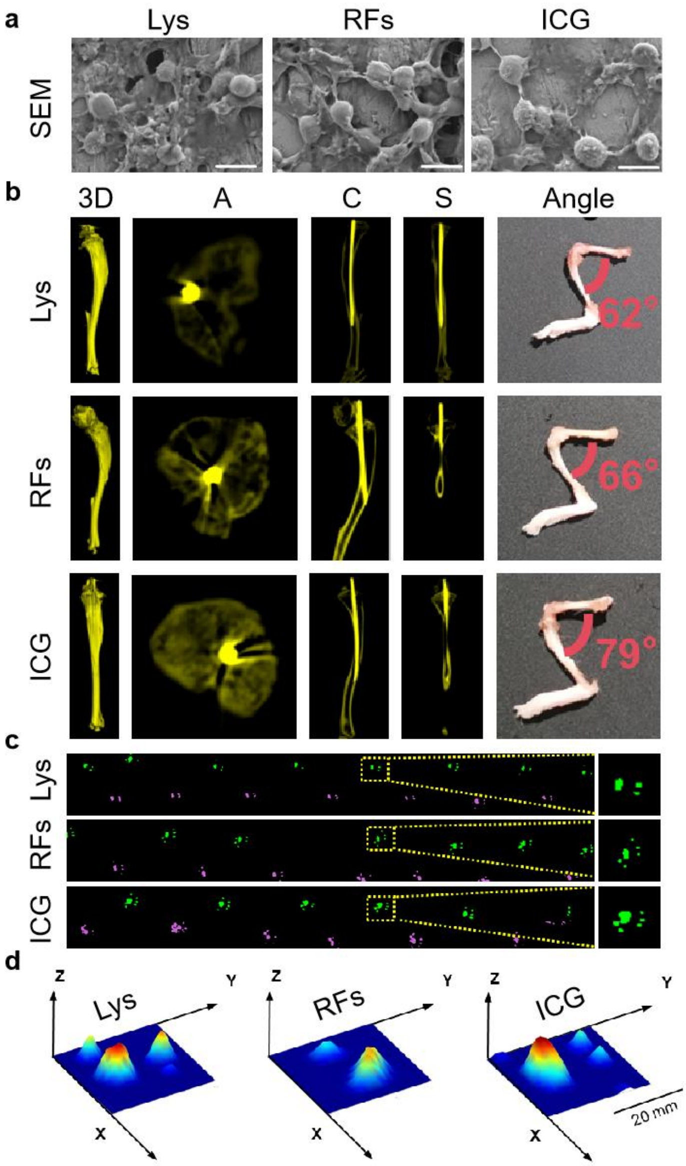

Supplementary Fig. 32 | *In-vivo* therapeutic efficacy evaluation of different hydrogels in a MRSA-infected prosthetic joint implants (PJI) murine model. **a,** The SEM images of MRSA biofilm on the surface of knee prosthesis at day 14 in various treated groups. Scale bar: 1 μm. **b,** Axial, coronal, sagittal, and 3D reconstruction micro-CT images 4 weeks after MRSA infection in different treatment groups. The mouse tibias are shown in dark yellow, and the implant is shown in light yellow. **c,** Actual trajectory of the mice as they walked and images of the mouse footprints in different groups. Green, left hind footprints; Pink, right hind footprints. **d,** 3D reconstruction of left hind footprints of mice in different groups. The color bar indicates the pressure intensity of each part of the mouse's footprints on the ground.

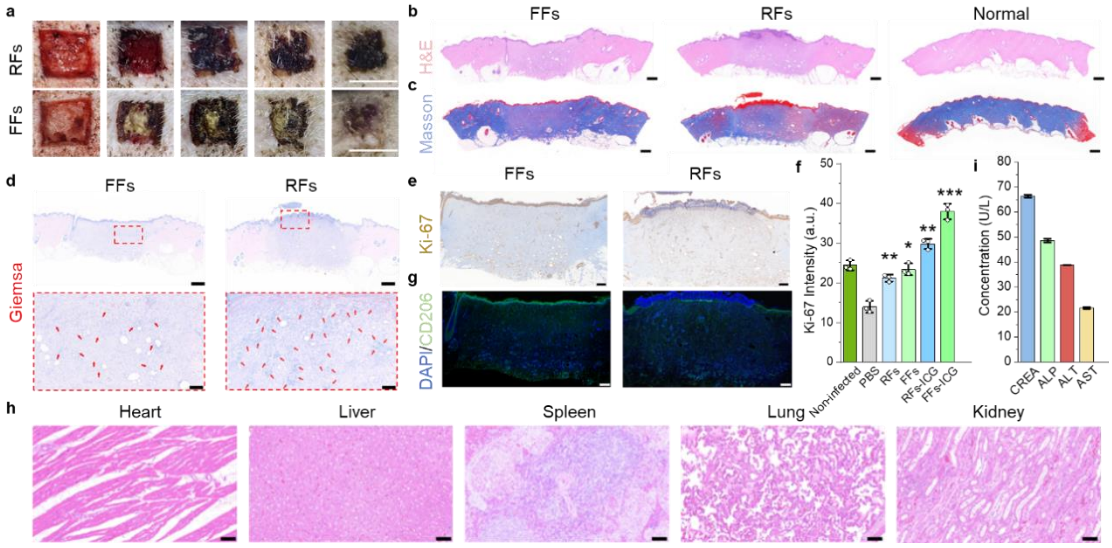

Supplementary Fig. 33 | *In-vivo* therapeutic efficacy evaluation of different hydrogels in a MRSA-infected porcine wound model. **a,** The representative macroscopic wound images of MRSA-infected Bama pig after various treatments (Scale bar: 1.5 cm). **b,** The representative H&E staining images of porcine wounds for the analysis of dermis and epidermis regeneration in different treatment groups on day 15. Scale bar: 1 mm. **c,** The representative Masson-staining images of porcine wounds for the analysis of collagen deposition in different treatment groups on day 15. Scale bar: 1 mm. **d,** The representative Giemsa-staining images of porcine wounds for the analysis of survival bacteria in different treatment groups on day 15. Scale bar: 1 mm (top), 200 μm (down). **e,** The representative Ki-67-staining images of porcine wounds for the analysis of cell proliferation in different treatment groups on day 15. Scale bar: 500 μm. **f,** Quantitative analysis of Ki-67 intensity (n = 3; data are presented as individual points; Non-infected ($p = 0.0100$), RFs ($p = 0.0091$), FFs ($p = 0.0145$), RFs-ICG ($p = 0.0050$), FFs-ICG ($p = 0.0010$) *vs.* PBS). **g,** The representative DAPI/CD206-staining images of porcine wounds for the analysis of macrophage polarization in different treatment groups on day 15. Scale bar: 500 μm. **h,** The representative H&E staining images of major organs (heart, liver, spleen, lung, and kidney) from MRSA-infected porcine model for the *in-vivo* biosafety characterization in different treatment groups on day 15. Scale bar: 50 μm. **i,** Blood biochemistry analysis (CREA, ALP, ALT, and AST) of MRSA-infected Bama pig on day 15 after the various treatments (n = 3). The data were expressed as mean ± standard deviation (S.D). Statistical significance was analyzed by one-way ANOVA using GraphPad Prism 8, followed by Tukey's post-hoc test for pairwise comparisons. Statistical significance was defined as *$p < 0.05$, **$p < 0.01$, and ***$p < 0.001$ *vs.* PBS. Source data are provided as a Source Data file.

Table S1. Primers used in this paper.

**Gene**	**Primer direction**	**Primer sequence (5'→3')**
Vegfa	Forward	AGGGCAGAATCATCACGAAGT
Vegfa	Reverse	AGGGTCTCGATTGGATGGCA
Hif1a	Forward	GAACGTCGAAAAGAAAAGTCTCG
Hif1a	Reverse	CCTTATCAAGATGCGAACTCACA
Il10	Forward	CCCATTCCTCGTCACGATCTC
Il10	Reverse	TCAGACTGGTTTGGGATAGGTTT
Pdgfb	Forward	CTCGATCCGCTCCTTTGATGA
Pdgfb	Reverse	CGTTGGTGCGGTCTATGAG

Supplementary References

1. Usov I, Mezzenga R. FiberApp: An Open-Source Software for Tracking and Analyzing Polymers, Filaments, Biomacromolecules, and Fibrous Objects. *Macromolecules* 2015, **48**(5)**:** 1269-1280.
2. Zhou J, Li T, Peydayesh M, Usuelli M, Lutz-Bueno V, Teng J*, et al.* Oat Plant Amyloids for Sustainable Functional Materials. *Adv. Sci (Weinh)* 2022, **9**(4)**:** e2104445.
3. Zhang W, Xuan Q, Zhang Q, Wang T, Wang C, Li H*, et al.* Near-infrared light switching nitric oxide nanogenerator with "linkage mechanism" for tumor targeting multimodal synergistic therapy. *Sci. China:Chem.* 2022, **66**(2)**:** 586-600.
4. Lv J, Qiu Y, Pan L, Zhang X, Li M, Yin X. Photothermal/photodynamic antibacterial hydrogel embedded with copper carbon dots and Au nanoparticles. *Nano TransMed* 2024, **3:** 100034.
5. Jiang F, Wang J, Ren Z, Hu Y, Wang B, Li M*, et al.* Targeted Light-Induced Immunomodulatory Strategy for Implant-Associated Infections *via* Reversing Biofilm-Mediated Immunosuppression. *ACS Nano* 2024, **18**(9)**:** 6990-7010.
6. Xuan Q, Jiang F, Dong H, Zhang W, Zhang F, Ma T*, et al.* Bioinspired Intrinsic Versatile Hydrogel Fabricated by Amyloidal Toxin Simulant-Based Nanofibrous Assemblies for Accelerated Diabetic Wound Healing. *Adv. Funct. Mater.* 2021, **31**(49)**:** 2106705.
7. Xuan Q, Cai J, Gao Y, Qiao X, Jin T, Peydayesh M*, et al.* Amyloid-Templated Ceria Nanozyme Reinforced Microneedle for Diabetic Wound Treatments. *Adv. Mater.* 2025**:** e2417774.
8. Zhong W, Meng H, Ma L, Wan X, Chen S, Ma K*, et al.* Hydrogels loaded with MSC-derived small extracellular vesicles: A novel cell-free tissue engineering system for diabetic wound management. *VIEW* 2024, **5**(4)**:** 20230110.
