# Supplementary material for: Photo-reversible amyloid nanoNETs for regenerative antimicrobial therapies
Source: Nat Commun. 2025 Dec 10;16:11025. doi: 10.1038/s41467-025-65976-6 (PMC12696075; doi:10.1038/s41467-025-65976-6)
Supplement: Supplementary file 2 — Reporting Summary [file 41467_2025_65976_MOESM2_ESM.pdf]

nature portfolio

Corresponding author(s): Chao Chen, Jiangtao Zhou, Raffaele Mezzenga

Last updated by author(s): Oct 7, 2025

Reporting Summary

Nature Portfolio wishes to improve the reproducibility of the work that we publish. This form provides structure for consistency and transparency in reporting. For further information on Nature Portfolio policies, see our Editorial Policies and the Editorial Policy Checklist.

Statistics

For all statistical analyses, confirm that the following items are present in the figure legend, table legend, main text, or Methods section.

n/a	Confirmed	
☐	☒	The exact sample size (*n*) for each experimental group/condition, given as a discrete number and unit of measurement
☐	☒	A statement on whether measurements were taken from distinct samples or whether the same sample was measured repeatedly
☐	☒	The statistical test(s) used AND whether they are one- or two-sided<br>*Only common tests should be described solely by name; describe more complex techniques in the Methods section.*
☒	☐	A description of all covariates tested
☒	☐	A description of any assumptions or corrections, such as tests of normality and adjustment for multiple comparisons
☐	☒	A full description of the statistical parameters including central tendency (e.g. means) or other basic estimates (e.g. regression coefficient) AND variation (e.g. standard deviation) or associated estimates of uncertainty (e.g. confidence intervals)
☐	☒	For null hypothesis testing, the test statistic (e.g. *F*, *t*, *r*) with confidence intervals, effect sizes, degrees of freedom and *P* value noted<br>*Give P values as exact values whenever suitable.*
☒	☐	For Bayesian analysis, information on the choice of priors and Markov chain Monte Carlo settings
☒	☐	For hierarchical and complex designs, identification of the appropriate level for tests and full reporting of outcomes
☒	☐	Estimates of effect sizes (e.g. Cohen's *d*, Pearson's *r*), indicating how they were calculated

Our web collection on statistics for biologists contains articles on many of the points above.

Software and code

Policy information about availability of computer code

Data collection	Nanoscope 8.1 (Bruker, USA); inductively coupled plasma mass spectrometry (ICP-MS, Agilent 8900, USA); ultraviolet/visible spectrophotometry (UV/Vis, UV-5100 Japan Hitachi); micro-computed tomography (micro-CT) analysis (Skyscan 1172, Bruker Micro-CT, Germany);
Data analysis	Nanoscope Analysis 8.1 (Bruker, USA); FiberApp (home made); SpectraManager (Jasco, Japan); micro-computed tomography (micro-CT) analysis (Skyscan 1172, Bruker Micro-CT, Germany); CTAn software (Skyscan, Bruker Micro-CT, Germany)

For manuscripts utilizing custom algorithms or software that are central to the research but not yet described in published literature, software must be made available to editors and reviewers. We strongly encourage code deposition in a community repository (e.g. GitHub). See the Nature Portfolio guidelines for submitting code & software for further information.

Data

Policy information about availability of data

All manuscripts must include a data availability statement. This statement should provide the following information, where applicable:

- Accession codes, unique identifiers, or web links for publicly available datasets
- A description of any restrictions on data availability
- For clinical datasets or third party data, please ensure that the statement adheres to our policy

All data that support the findings of this study are available within the article and the Supplementary Information. Source data are provided with this paper.

Research involving human participants, their data, or biological material

Policy information about studies with human participants or human data. See also policy information about sex, gender (identity/presentation), and sexual orientation and race, ethnicity and racism.

Reporting on sex and gender	n.a.
Reporting on race, ethnicity, or other socially relevant groupings	n.a.
Population characteristics	n.a.
Recruitment	n.a.
Ethics oversight	n.a.

Note that full information on the approval of the study protocol must also be provided in the manuscript.

Field-specific reporting

Please select the one below that is the best fit for your research. If you are not sure, read the appropriate sections before making your selection.

☒ Life sciences ☐ Behavioural & social sciences ☐ Ecological, evolutionary & environmental sciences

For a reference copy of the document with all sections, see nature.com/documents/nr-reporting-summary-flat.pdf

Life sciences study design

All studies must disclose on these points even when the disclosure is negative.

Sample size	All experiments (exept the porcine model) were repeated with more than three independent samples (data are presented in the figures as individual points). For porcine model, multiple (18) square dermal wounds (1.5 cm × 1.5 cm × 0.6 cm) were bilaterally created on the porcine dorsum, assigned to various treatment groups with three wounds per group.
Data exclusions	No data were excluded.
Replication	Reproducibility was confirmed by triplicated experiments/wounds.
Randomization	All experiments (expect the porcine model) were independent experiments and for porcine model, the assignment of 18 wounds to various groups was arranged in a randomized and crosswise manner.
Blinding	The blinding is not possible as the wounds in mice and pig have to be treated with distinct materials.

Reporting for specific materials, systems and methods

We require information from authors about some types of materials, experimental systems and methods used in many studies. Here, indicate whether each material, system or method listed is relevant to your study. If you are not sure if a list item applies to your research, read the appropriate section before selecting a response.

Materials & experimental systems

n/a	Involved in the study	
☒	☐	Antibodies
☐	☒	Eukaryotic cell lines
☒	☐	Palaeontology and archaeology
☐	☒	Animals and other organisms
☒	☐	Clinical data
☒	☐	Dual use research of concern
☒	☐	Plants

Methods

n/a	Involved in the study	
☒	☐	ChIP-seq
☒	☐	Flow cytometry
☒	☐	MRI-based neuroimaging

Eukaryotic cell lines

Policy information about cell lines and Sex and Gender in Research

Cell line source(s)	All cell lines including Raw 264.7 (TCM13), HUVEC, and L-929 (GNM28) cells were acquired from the Cell Bank of the Shanghai Institutes for Biological Sciences, Chinese Academy of Sciences.
Authentication	The identity of the all these cell line was confirmed by short tandem repeat (STR) profiling, and the epithelial characteristics were verified by positive immunofluorescence staining. These authentications were performed by the Cell Bank of the Shanghai Institutes for Biological Sciences, Chinese Academy of Sciences.
Mycoplasma contamination	All cell lines were routinely tested for mycoplasma contamination using a PCR-based assay and were confirmed to be negative. The Cell Bank of the Shanghai Institutes for Biological Sciences, Chinese Academy of Sciences confirmed that.
Commonly misidentified lines (See ICLAC register)	n.a.

Animals and other research organisms

Policy information about studies involving animals; ARRIVE guidelines recommended for reporting animal research, and Sex and Gender in Research

Laboratory animals	Mice are six-week-old healthy BALB/c mice; The pig is a female Bama pig (~25 kg weight).
Wild animals	n.a.
Reporting on sex	no sex based analysis.
Field-collected samples	n.a.
Ethics oversight	All animal experiments were conducted in accordance with Chinese legislation on the Use and Care of Research Animals (Document No. 55, 2001), and institutional guidelines for the Care and Use of Laboratory Animals established by the Shanghai University Animal Studies Committee, and this committee approved the experiments (ECSHU 2025-014).

Note that full information on the approval of the study protocol must also be provided in the manuscript.

Plants

Seed stocks	n.a.
Novel plant genotypes	n.a.
Authentication	n.a.
